# *In vitro* study on the mechanical strength of abutment screw head under compressive forces

**DOI:** 10.6026/973206300214198

**Published:** 2025-11-15

**Authors:** Sunita Chaudhary, Preeti Bhadouria, Deepak Tomar, Meenakshi Singh Tomar, Rushit Patel, Amit Kumar Upadhyay

**Affiliations:** 1 Department of Prosthodontics, DJ College of Dental Sciences and Research, Modinagar, Uttar Pradesh, India; 2 Department of Oral Medicine & Radiology, Maharana Pratap college of Dentistry & Reasearch Centre, Gwalior, Madhya Pradesh, India; 3 Department of Orthodontics, Ram Krishna Dharmarth Foundation Dental College and Research Centre Bhopal, Madhya Pradesh, India; 4 Department of Prosthodontics, Ram Krishna Dharmarth Foundation Dental College and Research Centre Bhopal, Madhya Pradesh, India; 5 Department of Oral & Maxillofacial Surgery, Karnavati School of Dentistry, Karnavati University, Gandhinagar, Gujrat, India; 6 Department of Periodontology, Shri Balaji Institute of Dental Sciences, Raipur, Chhattisgarh, India

**Keywords:** Dental implants, abutment screw, mechanical strength, compressive force, screw head design, *In vitro* study

## Abstract

The biomechanical stability of implant-abutment connections is influenced by abutment screw head design. Therefore, it is of interest to compare fracture resistance, deformation and torque loss among hexagonal, star-shaped and conical screw head interfaces. Forty-five titanium assemblies were loaded axially until failure and outcomes were analyzed with ANOVA and Tukey's test. Conical screws showed significantly higher fracture resistance, lower deformation and better preload retention than other designs (p < 0.001). Thus, we show that conical interface screws provide superior mechanical stability under compressive forces.

## Background:

The use of endosseous dental implants has become a predictable and highly successful treatment modality for replacing missing teeth, restoring function and improving patient quality of life [1]. The long-term success of these restorations is contingent not only on osseointegration but also on the biomechanical stability and integrity of the prosthetic components [2]. The connection between the implant and the abutment is a critical interface and the abutment screw is the central component responsible for clamping these two parts together and maintaining the stability of the entire suprastructure [3]. Despite high success rates, mechanical complications involving the implant-abutment connection are frequently reported, with abutment screw loosening being the most common, followed by screw fracture [4, 5]. These complications can lead to micro-gaps at the interface, promoting bacterial leakage and potentially causing instability of the prosthesis, patient discomfort and costly, time-consuming repairs [6]. Such failures are often attributed to the complex and high-magnitude occlusal forces experienced in the oral cavity, which can exceed the mechanical limits of the components [7]. In response to these clinical challenges, implant manufacturers have focused on optimizing the design of the abutment screw to enhance its mechanical performance. Research has explored various factors, including screw material, thread design, surface coatings and preload application techniques [8, 9]. However, the geometry of the screw head and its corresponding driver interface is an area of growing interest. This interface is responsible for transmitting the tightening torque from the driver to the screw shank to achieve the necessary preload (clamping force) and for resisting the deformation caused by functional loading [10]. A recent study by Nourizadeh *et al.* (2025) indicated that traditional hexagonal screw head designs, while widely used, may be prone to stripping or "rounding" of the internal hex during torque application or removal, especially after being subjected to functional loads [11]. The sharp internal angles of a hexagonal socket can act as stress concentration points, potentially leading to plastic deformation and reduced resistance to fracture under excessive occlusal forces [12]. Consequently, alternative designs such as star-shaped (e.g., Torx-style) and conical interfaces have been introduced. These designs propose to offer a larger contact surface area between the driver and the screw head, theoretically allowing for more efficient torque transfer and more uniform distribution of stresses under load [13]. Therefore, it is of interest to evaluate the mechanical strength and stability of three distinct abutment screw head designs (conventional hexagonal, star-shaped and conical interface) by measuring their ultimate fracture resistance, head deformation and torque maintenance after being subjected to a static axial compressive load.

## Materials and Methods:

## Study design:

This was an *in vitro* comparative experimental study designed to assess the mechanical performance of three different abutment screw head designs under a static compressive load.

## Sample size and grouping:

A total of 45 implant-abutment-screw assemblies were used. A power analysis was performed based on preliminary data from similar studies, determining that a sample size of n=15 per group would provide 80% power to detect a clinically meaningful difference with an alpha of 0.05.

The samples were randomly allocated into three experimental groups:

[1] Group A (n=15): Abutment screws with a conventional internal hexagonal head design.

[2] Group B (n=15): Abutment screws with a star-shaped (Torx-style) internal head design.

[3] Group C (n=15): Abutment screws with a conical internal interface head design.

## Inclusion and exclusion criteria:

All components used in the study were from a single manufacturer to eliminate inter-system variability.

Inclusion criteria specified new, unused and sterile-packaged components:

[1] Titanium dental implants (4.1 mm diameter, 10 mm length).

[2] Standard titanium abutments compatible with the implants.

[3] Titanium alloy (Ti-6Al-4V) abutment screws corresponding to the three head designs.

Any component showing visual defects or damage upon inspection was excluded from the study.

## Sample preparation:

Each of the 45 dental implants was embedded vertically in a cylindrical block of auto-polymerizing epoxy resin (EpoxyFix, Struers ApS, Denmark) using a custom-made alignment jig to ensure the implant platform was parallel to the base of the block and perpendicular to the direction of the applied force. After the resin had fully cured (24 hours), the corresponding abutment for each sample was seated onto the implant. The designated abutment screw for each group was then inserted and tightened using a calibrated digital torque driver (Tohnichi, STC200CN, Japan). All screws were tightened to a preload torque of 30 Ncm, as recommended by the manufacturer. The tightening process was performed by a single, trained operator. The assemblies were then left undisturbed for 10 minutes to allow for the dissipation of settling effects.

## Mechanical testing procedure:

Each resin-mounted sample was secured in a custom stainless-steel holder and placed in a universal testing machine (Instron Model 5965, Instron Corp., USA) equipped with a 5 kN load cell. A static, axial compressive load was applied to the center of the abutment's occlusal surface through a 3-mm-diameter flat-ended stainless-steel cylindrical piston. The load was applied at a constant crosshead speed of 1 mm/min. The test was conducted until a clear fracture event was observed, defined as a sudden drop of 30% or more in the registered load on the load-displacement curve. The test was automatically stopped after this event.

## Outcome measurements:

Three parameters were recorded for each sample:

[1] Ultimate fracture resistance (N): The maximum load (in Newtons) registered by the universal testing machine just before the fracture event.

[2] Screw head deformation (µm): Before and after mechanical testing, the internal geometry of each screw head was imaged using a digital microscope (Keyence VHX-7000, Japan) at 200x magnification. Standardized measurements of the internal width of the driver engagement feature were taken. Deformation was calculated as the percentage change and absolute difference (in micrometers) between the pre- and post-loading measurements.

[3] Post-Loading removal torque value (RTV) (Ncm): After the compressive loading test, the same digital torque driver used for tightening was used to measure the torque required to loosen each screw. The peak torque value recorded during the initial 30 degrees of counter-clockwise rotation was defined as the RTV.

## Statistical analysis:

All collected data were entered into SPSS software (Version 26.0, IBM Corp., USA). The normality of the data distribution for each variable was confirmed using the Shapiro-Wilk test. Descriptive statistics, including mean and standard deviation (SD), were calculated for all three outcome measures in each group. A one-way analysis of variance (ANOVA) was used to compare the means among the three groups. If the ANOVA results were statistically significant, a Tukey's Honestly Significant Difference (HSD) post-hoc test was performed for pairwise comparisons between the groups. The level of statistical significance was set at p < 0.05.

## Results:

All 45 samples were successfully tested to failure. The comparison of ultimate fracture resistance among the three screw head designs (Table 1 - see PDF) revealed a statistically significant difference (p < 0.001). Group C (Conical Interface) exhibited the highest mean fracture resistance (962.5 ± 60.1 N), followed by Group B (Star-Shaped: 848.3 ± 52.7 N) and Group A (Hexagonal: 755.9 ± 45.2 N). Comparison of screw head deformation, quantified as the absolute change in internal driver width, also showed significant differences between groups (p < 0.001) (Table 2 - see PDF). Group A (Hexagonal) demonstrated the highest mean deformation (25.4 ± 4.1 µm), which was significantly greater than both Group B (18.2 ± 3.5 µm; p < 0.001) and Group C (12.8 ± 2.9 µm; p < 0.001). Group B also showed significantly more deformation than Group C (p < 0.01).

The post-loading removal torque value (RTV), representing the residual preload following cyclic compressive loading, differed significantly across groups (p < 0.001) (Table 3 - see PDF). Group C (Conical Interface) maintained the highest RTV (27.9 ± 1.9 Ncm), followed by Group B (25.1 ± 2.2 Ncm) and Group A (21.5 ± 2.8 Ncm). Post-hoc analysis revealed that Group C exhibited significantly higher RTV than both Group B (p < 0.01) and Group A (p < 0.001), while Group B showed significantly higher RTV than Group A (p < 0.001). Group C (Conical Interface) consistently demonstrated superior mechanical performance in all parameters-highest fracture resistance, least deformation, and greatest post-loading RTV-indicating enhanced structural integrity and torque retention compared with the Hexagonal and Star-Shaped designs.

## Discussion:

The findings of this *in vitro* study clearly demonstrate that abutment screw head design has a significant influence on the mechanical stability and failure resistance of the implant-abutment connection under compressive loading. The null hypothesis, which stated no difference in performance among the three designs, was rejected for all measured parameters. The conical interface screw head (Group C) consistently outperformed the star-shaped (Group B) and conventional hexagonal (Group A) designs in terms of fracture resistance, resistance to deformation and preload maintenance. The superior performance of the conical interface design can be attributed to its geometric properties. A conical engagement provides a larger surface contact area and a more intimate fit between the driver and the screw head. This geometry facilitates a more uniform distribution of both torsional stresses during tightening and compressive stresses during functional loading [13]. In contrast, the hexagonal design contains sharp internal line angles that act as stress concentration points [14]. Under high compressive loads, these areas are predisposed to plastic deformation and crack initiation, which explains the lower fracture resistance and higher deformation observed in Group A. The star-shaped design, with its increased number of contact points compared to the hexagonal design, represents an intermediate solution, offering better stress distribution than the hex but less than the continuous surface contact of the cone, which aligns with its intermediate performance in our results. These findings are consistent with finite element analysis studies that have shown lower stress peaks in conical connections compared to flat-to-flat or polygonal connections [15]. The measurement of post-loading RTV provides valuable insight into the maintenance of screw preload, which is the critical clamping force that prevents micromovement and loosening [16]. The significant drop in RTV for the hexagonal screws (Group A) suggests that substantial plastic deformation occurred at the screw head-driver interface during loading. This deformation, or "stripping," leads to a loss of structural integrity and a corresponding reduction in the stored elastic energy (preload) of the screw shank [17]. The conical interface screws (Group C) retained nearly 93% of their initial torque value, indicating minimal plastic deformation and superior maintenance of preload. This stability is clinically paramount, as preserved preload is directly correlated with a lower risk of screw loosening over time [8]. Our results build upon previous research that has largely focused on the torsional properties of screw heads or the fatigue resistance of the entire assembly. For example, a study found that Torx-style screws could withstand higher insertion torque before deformation compared to hexagonal screws, suggesting better torque transmission [10]. Our study complements this by demonstrating that the benefits of improved geometry extend to resisting occlusal-type compressive forces. The sequence of performance (Conical > Star-Shaped > Hexagonal) observed in our study provides a clear hierarchy of mechanical robustness under an overload scenario. Several limitations of this study must be acknowledged. First, as an *in vitro* investigation, it cannot fully replicate the complex biomechanical environment of the oral cavity, which includes thermal cycling, the corrosive effects of saliva and variable, multi-directional forces [2]. We applied only a static, axial compressive load, which represents a simplified "worst-case" overload scenario rather than the cumulative damage caused by cyclic fatigue loading. Future studies should incorporate fatigue testing to assess the long-term endurance of these screw head designs. Second, the study utilized components from a single implant system. While this enhances internal validity by eliminating inter-system variables, the results may not be generalizable to all implant systems, as manufacturing tolerances and material properties can differ. Finally, the role of the operator in tightening screws, though standardized, can introduce minor variability. Despite these limitations, the clinical implications of this study are significant. For patients in high-risk categories, such as those with bruxism or strong musculature, or for restorations in the posterior molar region, selecting an abutment screw with an optimized head design, such as a conical interface, could substantially reduce the risk of mechanical complications. The enhanced resistance to deformation and superior preload maintenance suggest a more durable and reliable connection, potentially leading to fewer maintenance appointments and improved long-term prosthetic survival.

## Conclusion:

Abutment screw head geometry plays a decisive role in the long-term stability of implant-supported restorations. Conical interface designs demonstrated superior strength, reduced deformation and better preload retention compared to hexagonal and star-shaped heads. Adopting such advanced designs may help minimize mechanical complications and improve clinical success rates.
